# Implication of ferroptosis in aging

**DOI:** 10.1038/s41420-021-00553-6

**Published:** 2021-06-28

**Authors:** Maryam Mazhar, Ahmad Ud Din, Hamid Ali, Guoqiang Yang, Wei Ren, Li Wang, Xiaohui Fan, Sijin Yang

**Affiliations:** 1 National Traditional Chinese Medicine Clinical Research Base, Affiliated Traditional Chinese Medicine Hospital, Southwest Medical University, Luzhou, China; 2 Cardiovascular and Cerebrovascular Research Center of Integrated Traditional Chinese and Western Medicine, Affiliated Traditional Chinese Medicine Hospital, Southwest Medical University, Luzhou, Sichuan China; 3 Drug Discovery Research Center, Southwest Medical University Luzhou, Luzhou, China; 4 Department of Biosciences, COMSATS University, Islamabad, Pakistan; 5 Department of Acupuncture and Rehabilitation, Affiliated Traditional Chinese Medicine Hospital, Southwest Medical University, Luzhou, Sichuan Province China; 6 Pharmaceutical Informatics Institute, College of Pharmaceutical Sciences, Zhejiang University, Hangzhou, Zhejiang China

**Keywords:** Chronic inflammation, Prognostic markers

## Abstract

Life is indeed continuously going through the irreversible and inevitable process of aging. The rate of aging process depends on various factors and varies individually. These factors include various environmental stimuli including exposure to toxic chemicals, psychological stress whereas suffering with various illnesses specially the chronic diseases serve as endogenous triggers. The basic underlying mechanism for all kinds of stresses is now known to be manifested as production of excessive ROS, exhaustion of ROS neutralizing antioxidant enzymes and proteins leading to imbalance in oxidation and antioxidant processes with subsequent oxidative stress induced inflammation affecting the cells, tissues, organs and the whole body. All these factors lead to conventional cell death either through necrosis, apoptosis, or autophagy. Currently, a newly identified mechanism of iron dependent regulated cell death called ferroptosis, is of special interest for its implication in pathogenesis of various diseases such as cardiovascular disease, neurological disorders, cancers, and various other age-related disorders (ARD). In ferroptosis, the cell death occur neither by conventional apoptosis, necrosis nor by autophagy, rather dysregulated iron in the cell mediates excessive lipid peroxidation of accumulated lethal lipids. It is not surprising to assume its role in aging as previous research have identified some solid cues on the subject. In this review, we will highlight the factual evidences to support the possible role and implication of ferroptosis in aging in order to declare the need to identify and explore the interventions to prevent excessive ferroptosis leading to accelerated aging and associated liabilities of aging.

## Aging—an inevitable life process

Life evolves as a continuous process of aging leading eventually to inevitable mortality. Aging is characterized by a gradual and progressive functional decline of physiological functions and organ systems with advancing time leading to the increased risk of debility, disease, and the end of lifespan [1, 2]. Aging is a complex interplay of various endogenous mechanisms that mainly involves the imbalance of pro and antioxidant levels [3], anabolism and catabolism [4, 5], imbalance in energy metabolism [6], and simultaneous stimulation of various immune responses. All these mechanisms together lead to mild pro-inflammatory states [7] and immune-senescence [2] that increase the risk of development of various diseases and associated debility which work as vicious cycle to favor further accelerated aging. The hallmarks of aging identified in different organisms, include genomic instability, telomere attrition, epigenetic alterations, loss of proteostasis, dysregulated nutrient sensing, mitochondrial dysfunction, cellular senescence, stem cell exhaustion, and altered intercellular communication [8]. However, still aging is not simple to explain and much remain to be explored.

## Evolving mechanisms of aging

The basic mechanism of aging is best described by the free radical theory by Denham Harman (1950s) that proposes aging to be a consequence of accumulation of oxidative damage from reactive oxygen species (ROS) generated as a by-product of various cellular metabolic processes, that increases with age and leads to shortening of lifespan [9]. Some newer concepts of biological imperfectness are evolving besides the more popular free radical theory of aging. The idea suggests that basis of aging underlies the fact that cells accumulate damage to macro molecules continuously from heterogeneity, imperfectness, and infidelity of biological systems over time during their lifespan leading to senescence. The time span for this biological imperfectness led senescence depends on the vigilant performance of metabolic and genetic systems for detection and correction of more severe damages leading to lesser accumulation of the milder forms of damage. More recently, it has been identified that ROS is not solely responsible for aging [10]. Thus, it is important to identify the origin and types of damage other than the free radicals, which are the most studied and well-known stressors, involved in initiating, maintaining and sustaining the aging mechanism [2].

## Aging, chronic inflammation, and chronic diseases

**Fig. 1:**
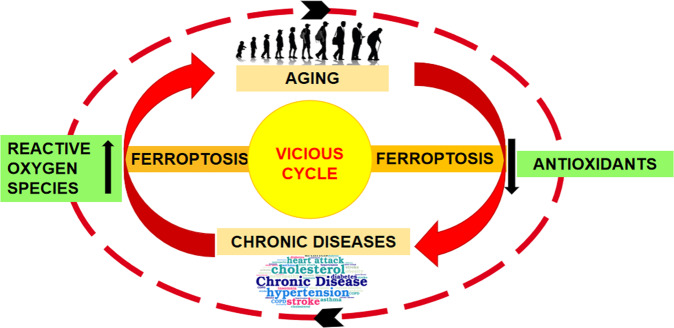
A vicious cycle of aging mechanism, promoted by underlying chronic diseases and ferroptosis. Illustration showing ferroptosis as a catalytic factor for induction of a vicious cycle of accelerated aging and chronic disease progression with underlying imbalance of oxidant and antioxidant defense mechanisms.

In the last decade, a new term “inflamm-aging” was coined in 2000 [11] to explain the process of aging as a chronic progressive rise in the pro-inflammatory status [12]. It is proposed that low-grade, asymptomatic, chronic, systemic, and non-resolving inflammation is present during inflamm-aging which serves as a determinant of the rate of the aging and lifespan. Previous scientific studies have described the concept of mild and constant chronic inflammation as a major risk factor underlying aging and related diseases such as atherosclerosis, arthritis, cancer, diabetes, osteoporosis, dementia, vascular diseases, obesity and metabolic syndrome [6]. As explained, aging is associated with redox imbalances with chronic induction and upregulation of pro-inflammatory mediators (e.g., TNF-α, IL-1β, IL-6, COX-2, iNOS) and signaling pathways such as NF-κB whereas diminution of activity of antioxidant pathways [7, 13]. So far, the cause and effect relationship between chronic diseases and aging bridged through the inflammation is still under investigation that is assumed to result in vicious cycle of accelerated frailty, aging and death. (Fig. 1)

## Role of iron and hepcidin in aging

**Fig. 2:**
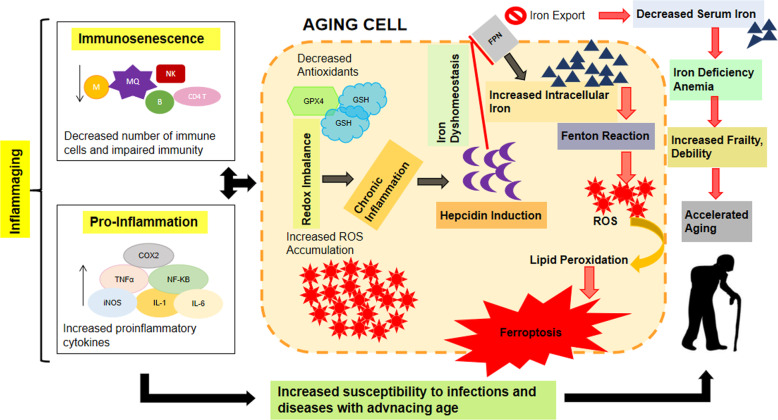
Proposed mechanism of interaction between iron dyshomeostasis, ferroptosis, and hepcidin in aging. Age related decline of immunity and redox imbalances leads to chronic inflammation and induction of hepcidin resulting in iron dyshomeostasis and ferroptosis that contributes to accelerated aging and age related frailty and debility.

Recently, the role of iron in accelerated aging has gained much focus of attention and is assumed to be the key player in accelerated aging. Aging is associated with imbalances of iron metabolism. Iron deficiency anemia is more prevalent in elderly population of age <80 with low serum ferritin and low systemic iron availability associated with detrimental consequences such as cardiovascular disease, increased falls and fractures, cognitive impairment, decreased quality of life, increased frailty, and higher risk of mortality [14]. Factors responsible for age related systemic iron depletion, other than poor diet and use of certain medication, may include elevated levels of circulating hepcidin due to underlying chronic inflammation [15]. However, further evidences are required to explore the effects of aging, iron status, and inflammation on regulation of hepcidin [14]. Iron stores in tissues are found to be increased with aging. This increased intracellular iron induces deleterious effects on cellular functions due to redox imbalances leading to ferroptosis that contributes to aging and associated morbidity and increased mortality [16]. The low systemic iron availability and increased intracellular iron levels may be linked with increased production of hepcidin due to underlying chronic inflammation associated with aging [15]. Therefore, viewing the above two strong facts with accumulating evidences favoring aging phenomenon, i.e., chronic inflammation and iron dyshomeostasis, hepcidin, could be expected to serve as a possible target of manipulation to gain benefit in controlling aging and related debility (Fig. 2).

Further, to answer some questions such as whether aging causes susceptibility to chronic diseases? Or does chronic diseases expose to early aging? We could focus on hepcidin, which is found to be increased in the elderly population as well as in chronic diseases leading to excess intracellular iron and ferroptosis [14, 15]. However, there is a lack of accumulated confirmatory studies regarding the role of hepcidin in ferroptosis and aging.

## Age-related dysegulation of iron and ferroptosis

All eukaryotes require iron for various biological processes such as energy production, DNA synthesis, replication, detoxification, etc. Iron is critical for growth and development of an organism, yet under strict regulatory mechanisms orchestrated by a set of transport, storage, and regulatory proteins to avoid its deficiency or accumulation with debilitating consequences [17, 18]. Despite tight regulatory mechanisms operating in a cell for iron homeostasis, there is no proper excretory mechanism [19] in our body except by hemorrhages or physiological peeling off of surface/luminal cells which remove as low as 1 mg of iron out of 4 g per day, with 1 mg of iron absorption by duodenum per day [19, 20]. Although, iron deficiency during growth and development causes deficits in major developmental pathways, but excessive iron retention in adult life leads to accelerated aging. The possible reasons for iron excess with aging include, (i) the decreased metabolic rate and need for iron by co-factor molecules; (ii) decreased amounts of hemoglobin, which contains 60% of total iron; and (iii) menopause induced relative iron overload in women. Excessive accumulation of iron in humans over a lifespan in somatic tissue induces deleterious effects on cellular functions leading to cell death and contributes to aging with associated morbidity and increased mortality [16]. It is supposed that this age related iron dyshomeostasis and subsequent cellular dysfunction might have a connection with a unique form of regulated cell death that depends on iron metabolism known as ferroptosis. However, accumulated supporting evidences are lacking.

Further, we can confer that iron is important for life and its regulation can keep cellular energetics in control whereas uncontrolled iron may lead to death via aggravation of disease pathogenesis and accelerated aging. Regulated iron in check, maintains energy and life whereas imbalances in iron regulation leads to energy imbalances and cytotoxicity. Given the plethora of disease implications, the identification of new modulators of iron metabolism and ferroptosis that can alleviate chronic diseases and retard accelerated aging could be a potential approach to healthy longevity.

## Ferroptosis—a unique mode of cell death

Conventionally, cell death occurs through necrosis, apoptosis, and autophagy [21]. Besides these modes of cell death, non-apoptotic mechanisms also exist [22] and that is ferroptosis. It is defined as iron-dependent regulated necrosis that is caused by massive lipid peroxidation-mediated membrane damage [23]. The role of ferroptosis in cardiovascular diseases, cancers and neurological diseases including aging has been identified [24, 25]. The term “ferroptosis” was first coined in 2012 after screening for small-molecule compounds capable of inhibiting the growth of RAS-mutant cancer cells [26]. The primary hypothetical idea of ferroptosis may have originated from nutrient reduction tempted cancer cell death [27] and “oxytosis”, which is the death of neurons succumbing to the excitotoxin glutamate and simultaneous inhibition of the amino acid antiporter solute carrier family 7 member 11 (SLC7A11/xCT/system xc^−^) [28, 29].

## Mechanism of ferroptosis

Ferroptosis is distinct from apoptosis and necroptosis as shown by its independent mediation in the absence of key effector of apoptosis and necroptosis i.e., BAX, BAK, caspase; mixed lineage kinase domain-like protein (MLKL), and receptor interacting serine/threonine kinases (RIPK1 and RIPK3) (ref. [6]). Although, ferroptosis has a cancer-protective effect on uncontrolled tumor cells, however, it is a most probable culprit underlying pathogenesis of various diseases and might be a hidden cause of many unidentified disease mechanisms [23, 30]. Despite rigorous research in the field of ferroptosis, still a lot remain to be explored to fully understand its mechanism and its role in the physiological and pathological conditions. So far, the mechanism of ferroptosis as described by various researchers can be simply comprised of four steps i.e., (i) inactivation of cysteine/glutathione antiporter system, (ii) depletion of glutathione and GPx4, (iii) excessive production of lipid ROS and (iv) excessive cellular iron accumulation [31–34]. The ferroptosis spreads in a paracrine manner via signals not clearly determined yet, but may include the toxic end-products of lipid peroxidation, 4-hydroxynonenal (4-HNE) and malondialdehyde (MDA) which are stable and react with biological macromolecules, up to distant sites from its origin [35]. Electron lucent nucleus is found to be a unique feature and hallmark of ferroptosis through transmission electron microscopy [36]. The morphological characteristics induced in a cell upon ferroptosis are distinct from apoptosis and mainly influence mitochondria. Dixon et al. observed mitochondrial shrinkage and dysfunction that indicate ferroptosis is directly linked with cellular energetics and mitochondrial dysfunction leading to alterations and lack of cellular energy and eventually cell death [26, 36].

## Oxidative stress, ferroptosis, and aging

Higher concentration of iron tempts ferroptosis by the production of ROS through the Fenton reaction. This leads to the conclusion that either increased iron uptake or iron storage may contribute to the process of ferroptosis [37, 38]. Suffice it to say that abnormalities in the metabolic system involved in iron acquisition and utilization are essential for the induction of ferroptosis. Excess iron, divalent ferrous ion Fe^2+^ precisely, can react with hydrogen peroxide (H_2_O_2_) or organic peroxide (ROOH) to yield soluble hydroxyl (HO) or lipid alkoxy (RO ∙ ) radicals, respectively. This is the primary source of reactive oxygen species (ROS) produced by Fe^2+^ in the cell, known as the Fenton reaction [37]. In addition aging is defined as organ and tissue failure with the advent of time through a systematic process [39]. Whereas oxidative stress leads to structural damage due to accumulation of biological molecules such as DNA, RNA, and proteins [40]. As previously described that aging is primarily affected by oxidative stress, it might be induced by ferroptosis; which leads to cell death [13]. In the case of aging, the cell’s normal ability to overcome oxidative stress goes on decline which ultimately leads to cellular degradation process [41]. In a similar way the cell normal antioxidant defense mechanism is unable to overcome the ferroptosis induced oxidative stress [42]. Therefore, it is anticipated that ferroptosis contributes to the process of aging.

The classic profile of ferroptosis is regulated cell death due to iron-dependent lipid peroxidation, which can be ameliorated by iron chelators and lipid antioxidants, and the culprit of lipid peroxidation is generally believed to be ROS that react with polyunsaturated fatty acids (PUFAs) of the membrane and induce lipid peroxidation. Several ROS producing routes have been proposed, however, the detailed mechanism of iron induced ROS remains unclear [43]. Some compounds that promote intracellular and mitochondrial ROS production, fail to promote iron-dependent cell death, but promote other types of regulatory cell death i.e., necrosis, apoptosis, etc [26, 44, 45]. An open question arises whether any type of lethal lipid peroxidation would be classified as ferroptosis, or specific types of lethal lipid peroxidation would be referred to as ferroptosis. It is, therefore, of great demand to obtain an understanding of the specific lipids and their precursors participating in ferroptosis.

## Inflammation, ferroptosis, and aging

As an important pathological process in various disease prognosis, inflammation has been a challenge for a long time [46]. ROS are the key players of inflammation triggered by various stimuli such as infection, tissue injury and other physical and chemical insults to the body [47]. The activity of nicotinamide adenine dinucleotide phosphate (NADPH) oxidases (NOXs) and the mitochondrial respiratory chain control the production of ROS which in turn promotes lipid peroxidation by arachidonate lipoxygenase (ALOX) or cytochrome P450 reductase (POR) [48]. Excessive peroxidation of phospholipid membranes rich in PUFAs is responsible for ferroptosis linked inflammation. Furthermore, all pathways of ferroptosis are associated with PUFAs in some way or the other [30, 49]. It is also clear that the process of aging cannot be separated from inflammation and oxidative stress [50]. The age related oxidative stress is related to imbalance between the glucocorticoid and inflammatory systems, which in turn exacerbates low grade immune activation [51]. Recently, ferroptosis is classified as a type of regulated necrosis where cell membranes rupture and release intracellular contents, especially damage-associated molecular patterns (DAMPs) [48]. An extensive body of evidence shows that ferroptosis is positively correlated with inflammation and many anti-inflammatory compounds also act as ferroptosis inhibitors [52] therefore, anti-inflammatory compounds might be helpful in controlling accelerated aging [53].

## Iron accumulation with aging is a conserved phenomenon

Iron is essentially required for various biochemical processes in our body during development. However, iron accumulates in the tissues with increasing age, pointing towards the incompetence of iron regulatory mechanisms that prevent abnormal redox cycling of iron, such as the Fe^2+^-glutathione complexes [54]. Moreover, this phenomenon of iron accumulation with aging is conserved and represents a remote consensus for susceptibility in late stages of life in many organisms including drosophila, nematodes, mammals, and humans [55–58]. Research is going on to prove the susceptibility of aged animals to ferroptotic death due to age-related increment of labile iron coupled with a reduction of glutathione levels implying that disruption to the iron-glutathione axis is fundamental to natural aging and death. Recently, researchers have proved in nematode model that ferroptosis is a universal and key feature which contribute and accelerate the process of aging [59].

## Examples of implication of iron and ferroptosis in aging

Aging is associated with increased risk of many diseases such as cancers, cardiovascular, cerebrovascular and neurodegenerative disorders [13, 60]. Aging has been associated with excess intracellular iron retention in many cell types, contributing to lipid peroxidation, aggregation of insoluble proteins in the endoplasmic reticulum (ER), damaging the DNA, and blocking genomic repair systems by downregulating p53, a process defined as ferrosenescence—premature aging phenotype. Thus, a vicious cycle sets up where aging associated excess iron retention induce genomic disintegration which in turn promotes further debilitation and aging by causing more DNA damage and DNA repair blockade. Combined with antioxidant failure, these changes may lead to cellular death by ferroptosis [61, 62].

Macular degeneration is associated with aging as GSH levels decrease and the retinal pigment is more prone to oxidative stress leading to senescence and death of retinal pigment epithelium and photoreceptors. Recent studies have found the direct link between GSH depletion and induction of ferroptosis, autophagy, and stress-induced premature senescence [63].

Excess iron supply is a lifespan hazard. Recent research shows that with increasing age, the cells in *Caenorhabditis elegans* undergo accumulation of ferrous iron. *C. elegans* undergo age-related gradual failure in capacity to safely sequester iron into ferritin and glutathione depletion, that trigger ferroptosis, with genetic effects, accelerated aging, and shortened lifespan. However, inhibiting the generation of lipid peroxides and limiting iron retention blocked the ferroptosis and decreased the age-related cell death with notable alleviation of health status and overall lifespan of the nematode [59]. Further, they observed increased lifespan and improved healthspan by inhibiting a cause of frailty i.e., ferroptotic death rather than by limiting the rate of aging. This concept could serve as potential therapeutic intervention which require further work to determine its effectiveness [59].

Neuronal iron retention induces DNA damage with genomic destabilization leading to premature aging [62]. In brain, iron levels rise with aging contributing to an age-dependent risk of ferroptosis which is associated with increased debility and neurodegeneration as seen in PD and AD [16]. The role of ferroptosis in neurodegeneration has been studied widely. Genetic evidence includes hippocampal neuronal loss and astrogliosis in GPx4 knockout adult AD mice [23]. Therefore, it is important to explore the post-developmental intervention strategies to control ferroptosis that may promote healthy aging.

## Link between ferroptosis and epigenetics in aging

**Fig. 3:**
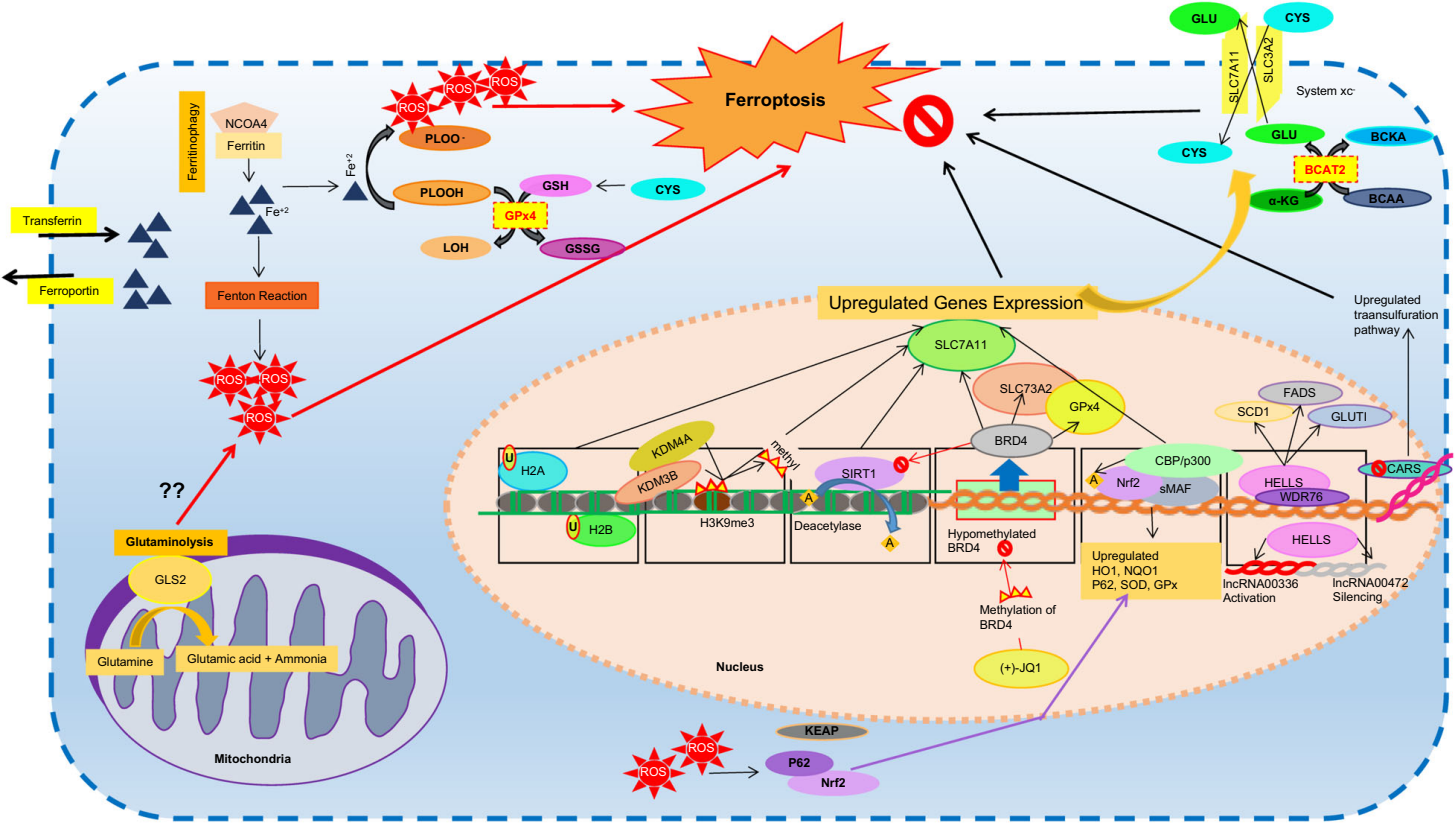
Epigenetic regulation of ferroptosis. Intracellular mechanism of ferroptosis regulated at various levels. In mitochondria, glutaminolysis increase ROS production via unkown mechanism and contributes to ferroptosis. At nuclear level, histone modifications such as ubiquitination, demethylation, or deacetylation; DNA transcriptional regulation via hypomethylation at specific loci, transcriptional factors, lncRNA; and metabolic regulation through proteins; inhibit ferroptosis.

Aging and related diseases are strongly linked to oxidative stress and inflammation with altered metabolic status and energy expenditure, which are known to be controlled by epigenetic mechanisms [64]. In a recent study, aging lens epithelial cells and that of cataracts are found to manifest higher redox imbalances, excessive production of reactive oxygen species, intracellular labile iron, and lipid peroxidation which are the hallmarks of ferroptosis. Transcriptome analysis in aged lens epithelial cells in both the human and mouse have revealed downregulation of genes associated with cellular redox and iron homeostases such as cystine/glutamate antiporter subunits SLC7A11 and SLC3A2 and iron exporter ferroportin (SLC40A1) [65]. Recently, accumulated evidence has shown that genes associated with ferroptosis are also controlled by epigenetic mechanisms [66]. Epigenetic changes occur at various levels, including the age-dependent global reduction in heterochromatin, modification of histones, altered noncoding RNA expression, and site-specific pattern of DNA methylation. Diet and other environmental factors can influence and change epigenetic information. However, the precise understanding of such changes requires further research [67]. Some examples of epigenetic regulation of ferroptosis are discussed below (Fig. 3).

## Epigenetic regulation of ferroptosis via histone modification

Histone modifications sush as ubiquitination of histones, histone 2 A (H2Aub) and histone 2B (H2Bub1, monoubiquitinated histone H2B on lysine 20), are found to activate SLC7A11expression and inhibit ferroptosis [66, 68]. Histone demethylases KDM4A and KDM3B inhibit ferroptosis by demethylation of histone H3 lysine 9 (H3K9me3) of SLC7A11 following its upregulated expression [66, 69]. However, depletion or knockdown of KDM4A promotes ferroptosis, as observed in osteosarcoma cells [69]. Interestingly, acetylation of trancription factor, Nrf2, by histone acetyltransferase (HAT) p300/CBP (CREB-binding protein) leads to transcriptional activation of its target genes including SLC7A11, thereby inhibiting ferroptosis. However, failure of NFE2L2 acetylation by ARF reduce the SLC7A11 expression and promote ferroptosis [66, 70]. Similarly, inhibition of histone deacetylase SIRT1, regulated by bromodomain-containing (BRD) protein BRD4, protects from ferroptosis. Inhibition of BRD4 by (+)-JQ1, inhibit histone methyltransferase G9a or enhance the histone deacetylase SIRT1 to inhibit BRD4, downregulate GPx4, SLC7A11, SLC3A2 gene expression, regulate ferritinophagy, and induce ferrroptosis in cancer cells [71].

## Epigenetic regulation of ferroptosis via DNA methylation

DNA methylation level is the most promising indicator to estimate biological aging. DNA methylation level increases with aging at specific loci, however, genome undergoes hypomethylation globally [72]. As discussed above that BRD4 regulate ferroptosis, however, its expression is epigenetically regulated via DNA methylation. Cancer tissues were found to express higher levels of BRD4 as compared to normal tissues with reduced DNA methylation at specific BRD4 loci and reduced ferroptosis. It has been suggested that BRD4 inhibitor (+)-JQ1, enhanced BRD4 DNA methylation to induce ferritinophagy and ferrroptosis in cancer cells [71]. Based on the fact that hypermethylation at specific loci is the epigenetic feature of both aging and ferroptosis, we can presume that ferroptosis might be an important feature of aging.

## Epigenetic regulation of ferroptosis via long non-coding RNA

Alteration in Long Non-Coding RNA such as epigenetic silencing of cytosolic lncRNA LINC00472/P53RRA (long intergenic non-protein coding RNA 472) and upregulation of the nuclear lncRNA LINC00336 (long intergenic non-protein coding RNA 336) mediated by chromatin remodeling protein, HELLS/LSH (lymphoid specific helicase), inhibit ferroptosis [66]. Moreover, HELLS interact with WDR76 (WD repeat domain 76) to inhibit ferroptosis by activating metabolic genes, including glucose transporter 1 (GLUT1), and sterol-CoA desaturase 1 (SCD1), and fatty acid desaturase 2 (FADS2), that are also related to ferroptosis [73].

## Metabolic control of epigenetics in ferroptosis

Metabolic products are also critical for regulation of DNA and chromatin modifications. For example, α-ketoglutarate (αKG) is required for DNA and histone demethylation, folate and vitamins B6/B12 induced *S*-adenosylmethyonine (SAM) is a DNA methyl donor; and succinate and fumarate inhibit histone demethylases. Therefore, metabolic alterations can generate perturbations of the whole epigenome [74]. Recently, the role of amino acids on regulating ferroptosis has been identified. Using a high-throughput CRISPR/Cas9-based genetic screen in HepG2 hepatocellular carcinoma cells, branched-chain amino acid aminotransferase 2 (BCAT2) is found to be a novel suppressor of ferroptosis by mediating de novo intracellular glutamate synthesis in vitro. BCATs acts as a shuttle for branched chain amino acids and branched chain keto acids (BCAAs-BCKAs), leading to reduced intracellular α-ketoglutarate level, higher intracellular glutamate levels and inhibition of ferroptosis [75]. Cellular metabolic products, such as iron and ROS, also play important role in ferroptosis. Ferritinophagy promotes ferroptosis whereas p62- Keap1-Nrf2 system is known to be a central inhibitory pathway of ferroptosis [76, 77]. Gluatmine mediates ferroptosis via glutaminolysis through its specific metabolic enzymes glutaminases specially GLS2, although the exact mechanism is unclear [75]. Furthermore, silencing cysteinyl tRNA synthetase (CARS), an enzyme involved in charging of tRNAs with cysteine for protein translation, upregulates the transsulfuration pathway, such as heat shock factor binding protein 1/ heat shock factor 1 (HSF1-HSPB1) pathway, Mucin1 C-terminal subunit/x-cystine/glutamate transporter (MUC1-C/xCT) pathway which antagonizes ferroptosis, studied in various types of cancers [75, 78].

Based on the above evidences of epigenetic regulation of ferroptosis, we can identify different ways to promote healthy aging by targeting ferroptosis, that may hold immense potential to control accelerated aging, age related diseases and extend life span.

## Controlling ferroptosis for better longevity

Ferroptosis can be prevented by the enzymatic reaction of two major antioxidant systems involving glutathione peroxidase 4 (GPx4) that catalyzes the reduction of lipid peroxides in a glutathione-dependent reaction and the recently identified ferroptosis suppressor protein (FSP1) that catalyzes the regeneration of ubiquinone (Coenzyme Q10, CoQ10), which act as a lipid peroxyl radical trap [26, 79].

GSH dependent lipid peroxide repair systems can protect membranes from peroxidation damage. GPx4 is a member of the GPx family with a broader substrate preference that can directly catalyze the conversion of endogenous lipid hydroperoxides into innocuous lipid alcohols at the expense of the oxidation of two molecules of GSH (ref. [80, 81]). The supply of cysteine, a component of GSH, is dependent on the cysteine-glutamate antiporter (System xc^-^) or the trans sulfuration pathway, where, a decrease in cysteine-glutamate metabolism suppresses GPx activity [81]. When any of the key steps in the phospholipid metabolic pathway is impaired, phospholipid peroxidation is induced, promoting ferroptosis in cells. Behrouz Hassannia collated four ways to activate ferroptosis [82]. Class I ferroptosis-inducing compounds (FINs) trigger ferroptosis by depleting intracellular GSH through the inhibition of System xc^-^ or glutamate-cysteine ligase (GCL). For example, erastin, a System xc^-^ inhibitor, can inhibit cysteine import, leading to GSH depletion and inactivation of the phospholipid peroxidase GPx4 (ref. [26]). However, some cells take advantage of the transsulfuration pathway to biosynthesize cysteine from methionine under conditions of limited cysteine availability, therefore bypassing the requirement for cysteine import via System xc^-^. Consequently, these cells are resistant to ferroptosis induced by System xc^-^ inhibitors [83]. Class II FINs trigger ferroptosis by directly inhibiting and inactivating GPx4. Class III FINs indirectly inhibit and inactivate GPx4. Class IV FINs exert marked effects through iron overload or the excessive activation of heme oxygenase 1 (HMOX1), which catalyzes the degradation of heme and biliverdin to iron and thus enhances ferroptosis by increasing LIP [78, 84], however, HMOX1 can also act in a cytoprotective way, probably depending on the activation level [85]. The protective effect of HMOX1 is attributed to its antioxidant activity, while its toxic effect is due to the enhanced generation of ferrous iron. Thus, excessive HMOX1 upregulation could be cytotoxic, while moderate upregulation could be cytoprotective [86]. The canonical way to protect cells from ferroptosis has almost always involved GPx4, but this notion has been questioned recently. Ubiquinone (also named coenzyme Q10) is named for its wide distribution in cells; as a redox-active lipid. Ubiquinone is important for aerobic respiration, which generates ATP in mitochondria. The discovery that FIN56 induces ferroptosis by depleting ubiquinone led to the speculation that a ubiquinone-related cytoprotective mechanism independent of GPx4 exists [87]. This hypothesis was recently confirmed. Doll et al. and Bersuker et al. defined a gene encoding a protein called FSP1, ferroptosis suppressor protein 1. They found that FSP1 induces lipid peroxidation by complementing a reduced form of ubiquinone called ubiquinol, and the modulation of FSP1 in cells might have clinical relevance [78, 88], thus providing a new strategy for the treatment of ferroptosis-related diseases.

Lipophillic antioxidants such as vitamin E, ferroststin-1 (Fer-1), liproxstatin-1 (Lip-1) and potent bioactive polyphenols are effective inhibitors of ferroptosis [89, 90]. Vitamin E has shown protection against ferroptotic cell death *in-vitro* and in GPx4-knockout mice in-vivo [34]. Alpha-tocopherol hydroquinones, an endogenous metabolite of vitamin E, inhibits 15-LOX by reducing the non-heme iron in the enzyme from the active Fe3+ state to the inactive Fe²^+^ state, thus inhibiting ferroptosis [91]. Fer-1 and Lip-1 are highly effective radical-trapping antioxidants (RTAs) in lipid bilayers, and they are comparatively more potent than vitamin E at inhibiting lipid autoxidation [34].

Additionally, identification of the effective adaptive homeostasis and repair systems against stress responses such as Keap1-Nrf2 system [89] in minimizing the accumulation of oxidative damage might be valuable to extend the lifespan and better longevity [9, 92, 93].

## Targetting Ferroportin-1 to control aging

**Fig. 4:**
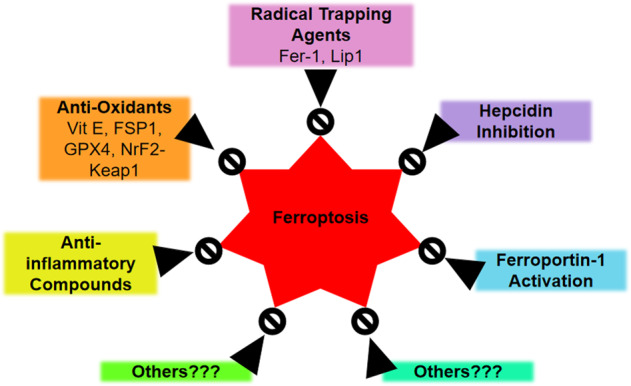
Illustration of possible therapeutic intervention targets to control ferroptosis induced accelerated aging. Besides the currently known classes of ferroptosis inhibitors such as radical trapping agents, anti-oxidants, anti-inflammatory agents, hepcidin inhibitors and ferropotin-1 activators, still there is a need of effective anti-ferroptotic compounds that could be used to prevent accelerated and debilitated aging.

Ferroportin1 (Fpn) is the only mammalian non-heme cellular iron exporter identified to date. It transports iron from iron storage cells and regulate systemic iron homeostasis [94]. A recent study demonstrated that loss of the iron exporter protein Fpn participates in the neuronal loss and memory impairment in neurological disorders [95]. A recent study confirmed that Fpn is the key factor in ferroptosis in mice aging model [96]. Recently it was found that knockdown of ferroportin accelerates erastin-induced ferroptosis [97, 98]. Thus, targeting Fpn could be a promising therapeutic strategy for neurodegenerative diseases including aging in the future [95] (Fig. 4).

## Conclusion

The role of iron has been widely studied in progression of multiple diseases, however, the role of ferroptosis in implication of various disorders is a recent topic of research since last decade. The excess iron retention associated with aging has also been studied widely but the generalized truth about implication of ferroptosis in aging is still a matter of considerable research. However, some researchers have already identified ferroptosis to be involved in accelerated aging. Therefore, interventions targeting ferroptosis as a course of aging and diseases may limit aging process and associated frailly and debility with better quality of life.

## Facts

- Aging is associated with chronic progressive rise in the pro-inflammatory state.
- Aging is associated with imbalance of iron metabolism and accumulation of iron stores in tissues which is a conserved phenomenon.
- Accumulated iron induces deleterious effects on cellular functions and contributes to aging by ferroptosis.

## Open Questions

- Could there be some triggers of aging other than reactive oxygen species?
- Whether ferroptosis is a silent hidden culprit of accelerated aging, frailty and debility?
- Could hepcidin become the target of intervention for reducing ferroptosis and chronic diseases for healthy aging?
- Is there any specific type of lethal lipid peroxidation involved in ferroptosis?
- Could epigenetic regulation of ferroptosis control accelerated aging?

## References

[1] Zhang G, Li J, Purkayastha S, Tang Y, Zhang H, Yin Y (2013). Hypothalamic programming of systemic ageing involving IKK-β, NF-κB and GnRH. Nature.

[2] Ponnappan S, Ponnappan U (2011). Aging and immune function: molecular mechanisms to interventions. Antioxid. Redox Signal..

[3] Zuo L, Zhou T, Pannell BK, Ziegler AC, Best T (2015). Biological and physiological role of reactive oxygen species−the good, the bad and the ugly. Acta Physiol..

[4] Rezuş E, Burlui A, Cardoneanu A, Rezuş C, Codreanu C, Pârvu M, et al. Inactivity and skeletal muscle metabolism: a vicious cycle in old age. Int J Mol Sci. 2020;21. 10.3390/ijms21020592 PMC7014434 31963330

[5] Roth SM, Metter EJ, Ling S, Ferrucci L (2006). Inflammatory factors in age-related muscle wasting. Curr Opin Rheumatol..

[6] Xia S, Zhang X, Zheng S, Khanabdali R, Kalionis B, Wu J (2016). An update on inflamm-aging: mechanisms, prevention, and treatment. Clin Dev Immunol..

[7] Chung HY, Cesari M, Anton S, Marzetti E, Giovannini S, Seo AY (2009). Molecular inflammation: underpinnings of aging and age-related diseases. Ageing Res Rev..

[8] López-Otín C, Blasco MA, Partridge L, Serrano M, Kroemer G (2013). The hallmarks of aging. Cell.

[9] Kirkwood TBL, Kowald A (2012). The free-radical theory of ageing−older, wiser and still alive: modelling positional effects of the primary targets of ROS reveals new support. Bioessays.

[10] Gladyshev VN (2014). The free radical theory of aging is dead. long live the damage theory. Antioxid Redox Signal..

[11] Franceschi C, Bonafè M, Valensin S, Olivieri F, Luca MD, Ottaviani E (2006). Inflamm‐aging: an evolutionary perspective on immunosenescence. Ann N Y Acad Sci..

[12] Ferrucci L, Fabbri E (2018). Inflammageing: chronic inflammation in ageing, cardiovascular disease, and frailty. Nat Rev Cardiol..

[13] Liguori I, Russo G, Curcio F, Bulli G, Aran L, Della-Morte D (2018). Oxidative stress, aging, and diseases. Clin Inter Aging.

[14] Dao MC, Meydani SN (2013). Iron biology, immunology, aging, and obesity: four fields connected by the small peptide hormone hepcidin. Adv Nutr..

[15] Fairweather-Tait SJ, Wawer AA, Gillings R, Jennings A, Myint PK (2014). Iron status in the elderly. Mech Ageing Dev..

[16] Toyokuni S, Yanatori I, Kong Y, Zheng H, Motooka Y, Jiang L (2020). Ferroptosis at the crossroads of infection, aging and cancer. Cancer Sci..

[17] Gems D, Partridge L (2013). Genetics of longevity in model organisms: debates and paradigm shifts. Annu Rev Physiol..

[18] Hare DJ, Arora M, Jenkins NL, Finkelstein DI, Doble PA, Bush AI (2015). Is early-life iron exposure critical in neurodegeneration?. Nat Rev Neurol..

[19] Coffey R, Ganz T (2017). Iron Homeostasis−an Anthropocentric Perspective. J. Biol. Chem..

[20] Muckenthaler MU, Rivella S, Hentze MW, Galy B (2017). A red carpet for iron metabolism. Cell.

[21] Galluzzi L, Maiuri MC, Vitale I, Zischka H, Castedo M, Zitvogel L (2007). Cell death modalities: classification and pathophysiological implications. Cell Death Differ..

[22] Tait SWG, Ichim G, Green DR (2014). Die another way – non-apoptotic mechanisms of cell death. J Cell Sci..

[23] Stockwell BR, Angeli JPF, Bayir H, Bush AI, Conrad M, Dixon SJ (2017). Ferroptosis: a regulated cell death nexus linking metabolism, redox biology, and disease. Cell.

[24] Yan H-F, Zou T, Tuo Q-Z, Xu S, Li H, Belaidi AA (2021). Ferroptosis: mechanisms and links with diseases. Signal Transduct Target Ther..

[25] Tang D, Chen X, Kang R, Kroemer G (2021). Ferroptosis: molecular mechanisms and health implications. Cell Res..

[26] Dixon SJ, Lemberg KM, Lamprecht MR, Skouta R, Zaitsev EM, Gleason CE (2012). Ferroptosis: an iron-dependent form of nonapoptotic cell death. Cell.

[27] Eagle H (1955). Nutrition needs of mammalian cells in tissue culture. Science.

[28] Tan S, Schubert D, Maher P (2001). Oxytosis: a novel form of programmed cell death. Curr Top Med Chem..

[29] Davis JB, Maher P (1994). Protein kinase C activation inhibits glutamate-induced cytotoxicity in a neuronal cell line. Brain Res..

[30] Yang WS, Stockwell BR (2016). Ferroptosis: death by lipid peroxidation. Trends Cell Biol..

[31] Cao JY, Dixon SJ (2016). Mechanisms of ferroptosis. Cell Mol Life Sci..

[32] Latunde-Dada GO (2017). Ferroptosis: role of lipid peroxidation, iron and ferritinophagy. Biochim Biophys Acta.

[33] Dixon SJ, Stockwell BR (2019). The hallmarks of ferroptosis. Annu Rev Cancer Biol..

[34] Kajarabille N, Latunde-Dada GO. Programmed cell-death by ferroptosis: antioxidants as mitigators. Int J Mol Sci. 2019;20. 10.3390/ijms20194968 PMC6801403 31597407

[35] Feng H, Stockwell BR (2018). Unsolved mysteries: how does lipid peroxidation cause ferroptosis?. PLoS Biol..

[36] Miyake S, Murai S, Kakuta S, Uchiyama Y, Nakano H (2020). Identification of the hallmarks of necroptosis and ferroptosis by transmission electron microscopy. Biochem Biophys Res Commun..

[37] Dixon SJ, Stockwell BR (2014). The role of iron and reactive oxygen species in cell death. Nat Chem Biol..

[38] Yagoda N, Rechenberg MV, Zaganjor E, Bauer AJ, Yang WS, Fridman DJ (2007). RAS–RAF–MEK-dependent oxidative cell death involving voltage-dependent anion channels. Nature.

[39] Flatt T (2012). A new definition of aging. Front Genet..

[40] Beckman KB, Ames BN (1998). The free radical theory of aging matures. Physiol Rev..

[41] Lemoine M (2020). Defining aging. Biol Philos..

[42] Yang WS, SriRamaratnam R, Welsch ME, Shimada K, Skouta R, Viswanathan VS (2014). Regulation of ferroptotic cancer cell death by GPX4. Cell.

[43] Tang S, Gao P, Chen H, Zhou X, Ou Y, He Y The Role of Iron, Its metabolism and ferroptosis in traumatic brain injury. Front Cell Neurosci. 2020;14. 10.3389/fncel.2020.590789 PMC7545318 33100976

[44] Skouta R, Dixon SJ, Wang J, Dunn DE, Orman M, Shimada K (2014). Ferrostatins inhibit oxidative lipid damage and cell death in diverse disease models. J Am Chem Soc..

[45] Su L-J, Zhang J-H, Gomez H, Murugan R, Hong X, Xu D (2019). Reactive oxygen species-induced lipid peroxidation in apoptosis, autophagy, and ferroptosis. Oxid. Med Cell Longev..

[46] Kearney CJ, Martin SJ (2017). An inflammatory perspective on necroptosis. Mol Cell..

[47] Wallach D, Kang T-B, Kovalenko A (2014). Concepts of tissue injury and cell death in inflammation: a historical perspective. Nat. Rev. Immunol..

[48] Kuang F, Liu J, Tang D, Kang R (2020). Oxidative damage and antioxidant defense in ferroptosis. Front Cell Dev Biol..

[49] Li J, Cao F, Yin H-l, Huang Z-j, Lin Z-t, Mao N (2020). Ferroptosis: past, present and future. Cell Death Dis..

[50] Wysocki K (2021). Genomics of aging: decreased immune defenses. J Am Assoc Nurse Pract..

[51] Petersen KS, Smith C (2016). Ageing-associated oxidative stress and inflammation are alleviated by products from grapes. Oxid Med Cell Longev..

[52] Sun Y, Chen P, Zhai B, Zhang M, Xiang Y, Fang J (2020). The emerging role of ferroptosis in inflammation. Biomed Pharmacother..

[53] Jones S, Anagnostou V, Lytle K, Parpart-Li S, Nesselbush M, Riley DR, et al. Personalized genomic analyses for cancer mutation discovery and interpretation. Sci Transl Med. 2015;7. 10.1126/scitranslmed.aaa7161 PMC4442685 25877891

[54] Berndt C, Lillig CH (2017). Glutathione, glutaredoxins, and iron. Antioxid Redox Signal..

[55] Pun PBL, Gruber J, Tang SY, Schaffer S, Ong RLS, Fong S (2010). Ageing in nematodes: do antioxidants extend lifespan in *Caenorhabditis elegans*?. Biogerontology.

[56] Vanhooren V, Libert C The mouse as a model organism in aging research: Usefulness, pitfalls and possibilities. Free Radic Biol Med. 2013;65. 10.1016/j.arr.2012.03.010 22543101

[57] Ward RJ, Zucca FA, Duyn JH, Crichton RR, Zecca L (2014). The role of iron in brain ageing and neurodegenerative disorders. Lancet Neurol..

[58] Dahiya R, Mohammad T, Alajmi MF, Rehman T, Hasan GM, Hussain A (2020). Insights into the conserved regulatory mechanisms of human and yeast aging. Biomolecules.

[59] Jenkins NL, James SA, Salim A, Sumardy F, Speed TP, Conrad M (2020). Changes in ferrous iron and glutathione promote ferroptosis and frailty in aging *Caenorhabditis elegans*. eLife.

[60] Niccoli T, Partridge L (2012). Ageing as a risk factor for disease. Curr Biol..

[61] Khan SS, Singer BD, Vaughan DE (2017). Molecular and physiological manifestations and measurement of aging in humans. Aging Cell..

[62] Sfera A, Bullock K, Price A, Inderias L, Osorio C (2018). Ferrosenescence: the iron age of neurodegeneration?. Mech Ageing Dev..

[63] Sun Y, Zheng Y, Wang C, Liu Y (2018). Glutathione depletion induces ferroptosis, autophagy, and premature cell senescence in retinal pigment epithelial cells. Cell Death Dis..

[64] Guzik TJ, Cosentino F (2018). Epigenetics and immunometabolism in diabetes and aging. Antioxid Redox Signal..

[65] Wei Z, Hao C, Huangfu J, Srinivasagan R, Zhang X, Fan X (2021). Aging lens epithelium is susceptible to ferroptosis. Free Radic Biol Med..

[66] Chen X, Li J, Kang R, Klionsky DJ, Tang D. Ferroptosis: machinery and regulation. Autophagy. 2020:1–28. 10.1080/15548627.2020.1810918 PMC8496712 32804006

[67] Pal S, Tyler JK (2016). Epigenetics and aging. Sci. Adv..

[68] Wang Y, Yang L, Zhang X, Cui W, Liu Y, Sun QR, et al. Epigenetic regulation of ferroptosis by H2B monoubiquitination and p53. EMBO Rep. 2019;20. 10.15252/embr.201847563 PMC6607012 31267712

[69] Chen M, Jiang Y, Sun Y (2021). KDM4A-mediated histone demethylation of SLC7A11 inhibits cell ferroptosis in osteosarcoma. Biochem Biophys Res Commun..

[70] Sun Z, Chin YE, Zhang DD (2009). Acetylation of Nrf2 by p300/CBP augments promoter-specific DNA binding of Nrf2 during the antioxidant response. Mol Cell Biol..

[71] Sui S, Zhang J, Xu S, Wang Q, Wang P, Pang D Ferritinophagy is required for the induction of ferroptosis by the bromodomain protein BRD4 inhibitor (+)-JQ1 in cancer cells. Cell Death Dis. 2019;10. 10.1038/s41419-019-1564-7 PMC6465411 30988278

[72] Jiang S, Guo Y (2020). Epigenetic clock: DNA methylation in aging. Stem Cells Int..

[73] Jiang Y, Mao C, Yang R, Yan B, Shi Y, Liu X (2017). EGLN1/c-Myc induced lymphoid-specific helicase inhibits ferroptosis through lipid metabolic gene expression changes. Theranostics.

[74] Cavalli G, Heard E (2019). Advances in epigenetics link genetics to the environment and disease. Nature.

[75] Wang K, Zhang Z, Hsiang-i T, Liu Y, Wang M, Song L (2020). Branched chain amino acid aminotransferase 2 regulates ferroptotic cell death in cancer cells. Cell Death Differ..

[76] Hou W, Xie Y, Song X, Sun X, Lotze MT, Zeh HJ (2016). Autophagy promotes ferroptosis by degradation of ferritin. Autophagy.

[77] Sun X, Ou Z, Chen R, Niu X, Chen D, Kang R (2016). Activation of the p62-Keap1-NRF2 pathway protects against ferroptosis in hepatocellular carcinoma cells. Hepatology.

[78] Hayano M, Yang WS, Corn CK, Pagano NC, Stockwell BR (2016). Loss of cysteinyl-tRNA synthetase (CARS) induces the transsulfuration pathway and inhibits ferroptosis induced by cystine deprivation. Cell Death Differ..

[79] Bersuker K, Hendricks JM, Li Z, Magtanong L, Ford B, Tang PH (2019). The CoQ oxidoreductase FSP1 acts parallel to GPX4 to inhibit ferroptosis. Nature.

[80] Ursini F, Maiorino M, Valente M, Ferri L, Gregolin C (1982). Purification from pig liver of a protein which protects liposomes and biomembranes from peroxidative degradation and exhibits glutathione peroxidase activity on phosphatidylcholine hydroperoxides. Biochim Biophys Acta.

[81] Deponte M (2013). Glutathione catalysis and the reaction mechanisms of glutathione-dependent enzymes. Biochim Biophys Acta.

[82] Wang W, Green M, Choi JE, Gijón M, Kennedy PD, Johnson JK (2019). CD8 + T cells regulate tumour ferroptosis during cancer immunotherapy. Nature.

[83] Hassannia B, Vandenabeele P, Berghe TV (2019). Targeting ferroptosis to iron out cancer. Cancer Cell..

[84] Chang LC, Chiang SK, Chen SE, Yu YL, Chou RH, Chang WC (2018). Heme oxygenase-1 mediates BAY 11-7085 induced ferroptosis. Cancer Lett..

[85] Hassannia B, Wiernicki B, Ingold I, Qu F, Herck SV, Tyurina YY (2018). Nano-targeted induction of dual ferroptotic mechanisms eradicates high-risk neuroblastoma. J Clin Invest..

[86] Sun X, Ou Z, Chen R, Niu X, Chen D, Kang R (2016). Activation of the p62‐Keap1‐NRF2 pathway protects against ferroptosis in hepatocellular carcinoma cells. Hepatology.

[87] Suttner DM, Dennery PA (1999). Reversal of HO-1 related cytoprotection with increased expression is due to reactive iron. FASEB J..

[88] Stockwell BR (2019). A powerful cell-protection system prevents cell death by ferroptosis. Nature.

[89] Doll S, Freitas FP, Shah R, Aldrovandi M, Silva MCD, Ingold I (2019). FSP1 is a glutathione-independent ferroptosis suppressor. Nature.

[90] Suzuki T, Yamamoto M (2017). Stress-sensing mechanisms and the physiological roles of the Keap1–Nrf2 system during cellular stress. J Biol Chem..

[91] Pomatto LCD, Davies KJA (2018). Adaptive homeostasis and the free radical theory of ageing. Free Radic Biol Med..

[92] Hinman A, Holst CR, Latham JC, Bruegger JJ, Ulas G, McCusker KP, et al. Vitamin E hydroquinone is an endogenous regulator of ferroptosis via redox control of 15-lipoxygenase. PLoS ONE. 2018;13. 10.1371/journal.pone.0201369 PMC6093661 30110365

[93] Kubben N, Zhang W, Wang L, Voss TC, Yang J, Qu J (2016). Repression of the antioxidant NRF2 pathway in premature. Aging Cell..

[94] Liu Z, Lv X, Song E, Song Y Fostered Nrf2 expression antagonizes iron overload and glutathione depletion to promote resistance of neuron-like cells to ferroptosis. Toxicol Appl Pharmacol. 2020;407. 10.1016/j.taap.2020.115241 32937103

[95] Zhang DL, Wu J, Shah BN, Greutélaers KC, Ghosh MC, Ollivierre H (2018). Erythrocytic ferroportin reduces intracellular iron accumulation, hemolysis, and malaria risk. Science.

[96] Bao WD, Pang P, Zhou XT, Hu F, Xiong W, Chen K, et al. Loss of ferroportin induces memory impairment by promoting ferroptosis in Alzheimer’s disease. Cell Death Differ. 2021:1–15. 10.1038/s41418-020-00685-9 PMC8166828 33398092

[97] Bao WD, Zhou XT, Zhou LT, Wang F, Yin X, Lu Y (2020). Targeting miR-124/Ferroportin signaling ameliorated neuronal cell death through inhibiting apoptosis and ferroptosis in aged intracerebral hemorrhage murine model. Aging Cell..

[98] Geng N, Shi BJ, Li SL, Zhong ZY, Li YC, Xua WL (2018). Knockdown of ferroportin accelerates erastin-induced ferroptosis in neuroblastoma cells. Eur Rev Med Pharm. Sci..

